# Paper-Based Versus Web-Based Versions of Self-Administered Questionnaires, Including Food-Frequency Questionnaires: Prospective Cohort Study

**DOI:** 10.2196/11997

**Published:** 2019-10-01

**Authors:** Itziar Zazpe, Susana Santiago, Carmen De la Fuente-Arrillaga, Jorge M Nuñez-Córdoba, Maira Bes-Rastrollo, Miguel Angel Martínez-González

**Affiliations:** 1 School of Pharmacy and Nutrition Department of Nutrition and Food Sciences and Physiology University of Navarra Pamplona Spain; 2 School of Medicine Department of Preventive Medicine and Public Health University of Navarra Pamplona Spain; 3 Instituto de Investigación Sanitaria de Navarra Pamplona Spain; 4 CIBER Fisiopatología de la Obesidad y Nutrición Instituto de Salud Carlos III Madrid Spain; 5 Department of Nutrition Harvard School of Public Health Boston, MA United States

**Keywords:** epidemiologic studies, cohort studies, surveys and questionnaires

## Abstract

**Background:**

Web-based questionnaires allow collecting data quickly, with minimal costs from large sample groups and through Web-based self-administered forms. Until recently, there has been a lack of evidence from large-scale epidemiological studies and nutrition surveys that have evaluated the comparison between traditional and new technologies to measure dietary intake.

**Objective:**

This study aimed to compare results from the general baseline questionnaire (Q_0) and the 10-year follow-up questionnaire (Q_10) in the Seguimiento Universidad de Navarra (SUN) prospective cohort, obtained from different subjects, some of whom used a paper-based version, and others used a Web-based version. Both baseline and 10-year assessments included a validated 136-item semiquantitative food-frequency questionnaire (FFQ), used to collect dietary intake.

**Methods:**

The SUN project is a prospective cohort study (with continuous open recruitment and many participants who were recently recruited). All participants were university graduates. Participants who completed the validated FFQ at baseline (FFQ_0, n=22,564) were selected. The variables analyzed were classified into 6 groups of questions: (1) FFQ (136 items), (2) healthy eating attitudes (10 items), (3) alcohol consumption (3 items), (4) physical activity during leisure time (17 items), (5) other activities (24 items), and (6) personality traits (3 items). Multiple linear and logistic regression models were used to assess the adjusted differences between the mean number of missing values and the risk of having apparently incorrect values for FFQ items or mismatches and inconsistencies in dietary variables.

**Results:**

Only 1.5% (339/22564) and 60.71% (6765/11144) participants reported their information using the Web-based version for Q_0 and Q_10, respectively, and 51.40 % (11598/22564) and 100.00% (11144/11144) of participants who completed the Q_0 and Q_10, respectively, had the option of choosing the Web-based version. Sociodemographic, lifestyle, health characteristics, food consumption, and energy and nutrient intakes were similar among participants, according to the type of questionnaire used in Q_10. Less than 0.5% of values were missing for items related to healthy eating attitudes, alcohol consumption, and personality traits in the Web-based questionnaires. The proportion of missing data in FFQ, leisure time physical activity, and other activities was higher in paper-based questionnaires than Web-based questionnaires. In Web-based questionnaires, a high degree of internal consistency was found when comparing answers that should not be contradictory, such as the frequency of fruit as dessert versus total fruit consumption and the frequency of fried food consumptions versus oil consumption.

**Conclusions:**

Incorporating a Web-based version for a baseline and 10-year questionnaire has not implicated a loss of data quality in this cohort of highly educated adults. Younger participants showed greater preference for Web-based questionnaires. Web-based questionnaires were filled out to a greater extent and with less missing items than paper-based questionnaires. Further research is needed to optimize data collection and response rate in Web-based questionnaires.

## Introduction

### Background

A key aspect in epidemiology is the adequate classification of exposure [1]. For this reason, the valid estimation of usual dietary intake in nutritional epidemiology studies is a topic of emerging interest, posing a complex and challenging task [2,3]. In general, the selection of the most appropriate instrument for assessing usual food intake in large-scale epidemiological studies depends on the research purpose. In this context, several dietary assessment methods are available, all of which have their own advantages and limitations [4]. There are 2 methods that prevail: the food-frequency questionnaire (FFQ), most frequently used in studies assessing the association between diet and health-related outcomes, and the 24-hour dietary recalls, primarily used in nutrition surveillance research [2]. Traditional approaches for gathering information from study participants include face-to-face or telephone interviews administered by trained dieticians, as well as paper or printed questionnaires, usually self-reported data [5,6]. These methods require a great deal of resources in terms of personnel, logistics, and materials [5,7]. However, in the past years, advances in technology and the wide use of the internet have allowed researchers to collect data quickly, with minimal costs from large sample groups, through the use of Web-based self-administered questionnaires [8-10]. It has been suggested that a Web-based FFQ can increase the response rate, which may result in greater validity of the data collected, compared with paper-based response rates [10]. However, most of the major limitations of conventional FFQs are similar to Web-based FFQs; therefore, the measurement errors in both approaches may remain essentially equivalent [11]. In fact, the cognitively complex completion process, inherent to the FFQ method (eg, averaging the usual frequency and portion size for each food group or specific food item), seems to be similar for paper-based and interactive computer-based or Web-based formats. The effectiveness of these types of data collection has previously been tested in different fields of health, several populations, and a variety of settings [12-18]. On the other hand, the integration of new technologies, including computer software and Web-based apps, has created novel and unique opportunities to conduct research focused on data collection in nutritional epidemiology [19]. In fact, there are several Web-based cohort studies in which the participants enroll via internet and all data are collected by Web-based questionnaires [5,7,17,20-23]. However, until now, there has been a scarcity of evidence from large-scale epidemiological studies and nutrition surveys that evaluate the comparison between traditional and new technologies to measure dietary intake. In the past 5 years, research in this area has attracted a growing interest [6,24]. The Seguimiento Universidad de Navarra (SUN) project offers an excellent opportunity to study this purpose, given that it is a large and well-known European cohort that uses both paper-based and Web-based FFQs, at baseline and/or during the follow-up, among participants with a high education level.

### Objectives

Therefore, we aimed to compare results from the general baseline questionnaire (Q_0) and the 10-year follow-up questionnaire (Q_10) of the SUN prospective cohort obtained in different subjects, some of whom used a paper-based version, and others used a Web-based version.

## Methods

### Study Population

**Figure 1:**
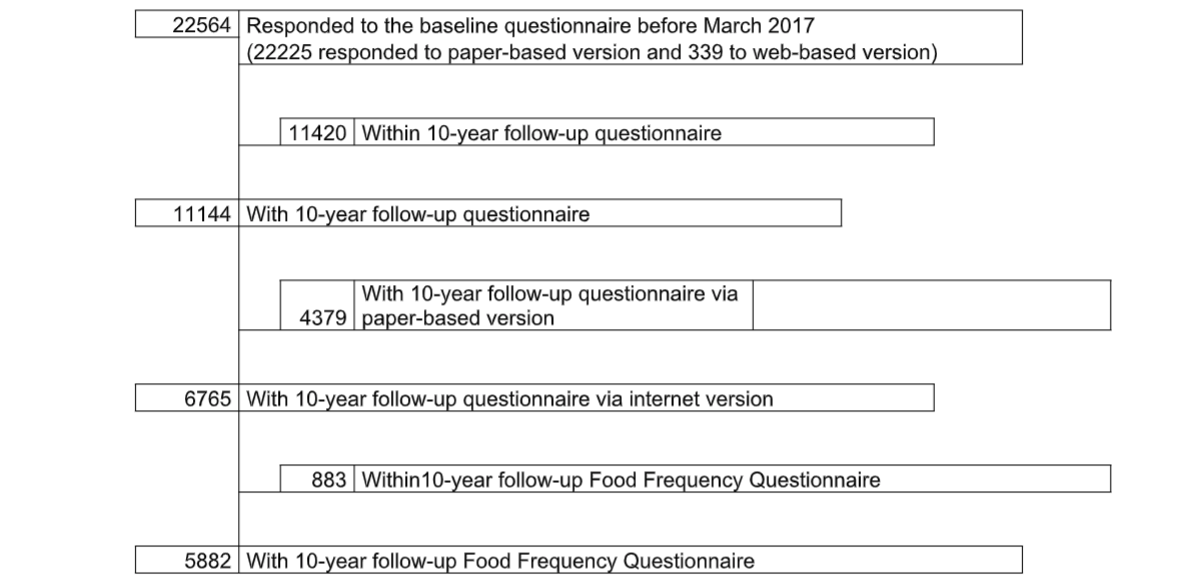
Flow chart of the study.

The SUN project is a multipurpose and prospective Spanish cohort of university graduates, designed to study the impact of several sociodemographic, nutrition, and lifestyle characteristics on the prevention of noncommunicable diseases. Open enrollment began in 1999. The design and methods used in the SUN project have been formerly described in detail elsewhere [25]. The study protocol was supported by the Institutional Review Board of the University of Navarra. We considered a response to the initial questionnaire as informed consent to participate. Participation in this cohort is only by invitation or registration. Currently, all questionnaires of the SUN cohort can be filled by paper or Web-based questionnaires. Web-based questionnaires were available since 2004, using a password-protected area of the SUN website. Starting at the beginning of 2004, participants were offered the possibility of answering their questionnaires either on the paper-based version or on the Web-based version. Thus, in each baseline or follow-up questionnaire since 2004, they can choose how to complete this questionnaire. For example, if a participant received the paper-based questionnaire, he or she can request his or her password to complete the Web-based version of the questionnaire on the SUN website. The participants who have access to the questionnaires do not necessarily have to complete all the questions. Diet in this cohort is evaluated through a repeatedly validated baseline semiquantitative FFQ [26,27]. At this time, the full-length FFQ has been administered only twice throughout the study, at baseline (FFQ_0) and after 10 years of follow-up (FFQ_10). Only those participants who have completed the Q_10 after 10 years on the internet can also fill the FFQ_10, although its filling is absolutely voluntary. For the present analysis, we used the latest available database from March 1, 2017, which included those participants who had responded to the Q_0. Of the 22,564 participants who completed the Q_0, 22,225 (98.5%) of them reported their data using the paper-based version; meanwhile, only 339 (1.5%) participants completed the Web-based version (see Figure 1). After a 10-year follow-up period, we collected questionnaires from 4379 (39.3%) participants by using the paper version and from 6765 (60.7%) participants by using the Web-based questionnaire. Among these 6765 participants who answered the 10-year questionnaire using the Web-based version, 5882 participants also completed a full-length FFQ via the internet (52.8% of the completed Q_10).

### Exposure Assessment

In the SUN cohort, the Q_0, and follow-up questionnaires are self-administered. The Q_0 includes information about sociodemographic variables (eg, sex, age, marital status, and employment status), lifestyle-related variables (eg, smoking status, physical activity, and special diets), anthropometric variables (weight, height), and clinical variables (medical history, family health history, blood pressure levels, medication use, and not gaining more than 5 kg of weight in previous years). On the other hand, the Q_10 collected information on a wide array of characteristics, including weight, height, marital status, and diagnosis of several diseases. Both of these general questionnaires can be filled out in paper form or on the Web, and they are exactly equivalent. The paper-based FFQ comprises 136 food and beverage items, categorized into 9 frequency categories of consumption. These items capture the usual consumption of listed foods during the previous year. There are 9 options for the average frequency of consumption (never or almost never: at least 6 times per day). The Web-based FFQ is a Web-based questionnaire, with a format very similar to that of the paper-based version including the same items.

### Measures of Data Quality

Nutrient intakes were calculated as the frequency multiplied by the nutrient composition of specified portion sizes for each food item by using an ad hoc computer program that was specifically developed for this aim. A trained dietitian updated the nutrient database using the latest available information from food composition tables for Spain [28,29]. We considered total energy intakes lower than 800 or 500 kcal/d for men and women, respectively, and greater than 4000 or 3200 kcal/d for men and women, as proposed by Willett as implausible energy intake [30]. In addition, we calculated the outliers of energy intake, defined as either percentiles 1 and 99 or 5 and 95. For each food group, the consumption was considered implausible if it fell outside the 25th percentile minus 3 times this interquartile range or the 75th percentile plus 3 times this interquartile range. For the sake of comparing the proportion of missing responses to the paper-based versus the Web-based questionnaires, we calculated the mean number of missing values in 6 sections of the baseline assessment of the cohort: (1) FFQ (136 items), (2) healthy eating attitudes questions (10 items), (3) alcohol consumption questions (3 items), (4) physical activity during leisure time questions (17 items), (5) other activities questions (24 items), and (6) personality traits questions (3 items). Finally, we evaluated differences in the following potential internal inconsistencies in reporting between paper-based and Web-based questionnaires: (1) eating fruit for dessert but reporting a total fruit consumption equal to 0, (2) eating fried foods at home but reporting olive oil consumption or other vegetable oil consumption equal to 0, (3) drinking alcohol sometimes but reporting total alcohol consumption equal to 0, and (4) drinking wine sometimes at lunch or dinner but reporting a total alcohol consumption equal to 0.

### Assessment of Other Variables

Physical activity information is obtained at baseline through a questionnaire validated in Spain [31], which collects information on 17 sports participated in, in the past year (with 10 answer options from never to more than 11 hours a week). The physical activity level during leisure time was quantified by assigning metabolic equivalents (METs) to each activity. Total METs-hours/week for each participant was calculated as the sum of the number of hours spent in each activity multiplied by the specific METs of that activity. Healthy eating attitudes are evaluated through 10 questions, asking the participants if they tried to eat more fruit, more vegetables, more fish, less meat, less sweets and pastries, more fiber and less fat, and if they tried to avoid the consumption of butter, removed fat from meat, and did not add sugar to drinks. We developed a score to capture the gathered information from these 10 questions, which was used in previous publications of the SUN cohort [32,33].

### Data Analysis

The aim of our study was not to compare repeated measurements of the same variables within the same subjects using 2 different methods; the aim of this study was to compare the reliability and comprehensiveness of the information gathered with each of both methods (paper-based or Web-based) from different subjects. Descriptive statistics were used to describe the differences at baseline and follow-up between participants who completed the questionnaire in paper-based version and among those who filled out the Web-based version. We used means and SDs for continuous variables or percentages for categorical variables. Multiple linear regression models were used to assess the association between the type of questionnaire (paper or Web-based version) and the differences in the mean number of missing values at baseline. Logistic regression models were run to assess the relationship between the type of questionnaire at baseline and the risk of having implausible data of food items and mismatches or inconsistencies in dietary variables. Both analyses were adjusted for sex, age, level of education (bachelor, graduate, postgraduate, and doctorate), and year entering the cohort (1999-2000, 2001, 2002-2003, 2004, 2005-2007, and 2008-2017). The Q_0 paper-based version was always considered as the reference category. Analyses were performed with STATA version 12 (STATA Corp). All *P* values are 2 tailed, and statistical significance was set at the conventional cut-off of *P*<.05.

## Results

### Baseline Characteristics

We have compared several potential measures of data quality between Web-based and paper-based data collection at baseline and at 10-year follow-up. Overall, the sociodemographic, lifestyle, and health characteristics were well balanced between the 2 approaches for administering the questionnaires, particularly Q_10, which had a higher number of participants and similar year of completion (Table 1).

Subjects who fulfilled the paper-based Q_0 were more likely to be older, married, workers, current smokers and ex-smokers, and they were more likely to have a lower level of university education. Moreover, they were less active and showed a higher prevalence of hypertension, cancer, diabetes, and weight gain in the past 5 years. On the other hand, participants who completed the Web-based Q_10 were more likely to be men, younger, physically active during leisure time, not married, workers, never smokers, had a higher adherence to the Mediterranean diet, and generally had less prevalence of chronic disease related to diet. Beneficial changes in the consumption of most food and macronutrients and a positive response to dietary attitudes were observed after 10 years of follow-up, mainly when comparing the paper FFQ_0 and FFQ_10 (Table 2).

**Table 1:** Baseline characteristics, mean (SDs), or percentages, of participants who filled out the paper- or Web-based questionnaires at baseline and at 10-year follow-up.

Variable		Baseline questionnaires (Q_0)		10-year of follow-up questionnaires (Q_10)	
		Paper-based (n=22,225)	Web-based (n=339)	Paper-based (n=4379)	Web-based (n=6765)
Age (years), mean (SD)		37.5 (12.4)	34.0 (11.7)	40.1 (12.8)	36.2 (10.9)
Men, n (%)		8592 (38.66)	129 (38.1)	1714 (39.14)	2786 (41.18)
Year of completing the questionnaire, mean (SD)		2004 (4)	2012 (4)	2002 (2)	2002 (2)
Body Mass Index (kg/m^2^), mean (SD)		23.5 (3.6)	23.6 (3.7)	23.6 (3.5)	23.4 (3.4)
**Marital status, n (%)**					
	Married	10973 (49.37)	108 (31.9)	2457 (56.11)	3336 (49.31)
	Single, widowed, divorced, and others	11252 (50.63)	231 (68.1)	1922 (43.89)	3429 (50.69)
**Occupation, n (%)**					
	Worker	17463 (78.57)	238 (70.2)	3419 (78.08)	5475 (80.93)
	Retired, housewife, and unemployed	4762 (21.43)	101 (29.8)	960 (21.92)	1290 (19.07)
Educational level (years of education), mean (SD)		5.0 (1.5)	5.5 (1.6)	5.0 (1.5)	5.0 (1.5)
Physical activity during leisure time (metabolic equivalents-h/week), mean (SD)		27.3 (24.3)	30.4 (25.5)	27.0 (24.2)	27.1 (23.1)
Mediterranean diet score (0 to 9 score), mean (SD)		4.3 (1.8)	4.5 (1.7)	4.2 (1.8)	4.1 (1.8)
TV (hours/week), mean (SD)		5.2 (2.1)	5.8 (2.0)	5.1 (2.0)	5.5 (2.0)
**Smoking status, n (%)**					
	Current smokers	4796 (21.57)	38 (11.2)	983 (22.45)	1500 (22.17)
	Ex-smokers	6202 (27.91)	74 (21.8)	1299 (29.66)	1872 (27.67)
	Never smokers	10597 (47.68)	224 (66.1)	2097 (47.89)	3303 (50.16)
Hypertension at baseline, n (%)		1933 (8.70)	21 (6.2)	437 (9.98)	483 (7.14)
Cancer at baseline, n (%)		843 (3.79)	12 (3.5)	175 (4.00)	232 (3.43)
Diabetes at baseline, n (%)		419 (1.89)	3 (0.9)	79 (1.8)	86 (1.27)
Dyslipemia at baseline, n (%)		1495 (6.73)	29 (8.6)	312 (7.1)	406 (6.00)
Cardiovascular disease at baseline, n (%)		335 (1.51)	7 (2.1)	70 (1.6)	80 (1.18)
Weight gain in past 5 years, n (%)		6692 (30.11)	93 (27.4)	1388 (31.70)	2086 (30.84)
Special diets, n (%)		1797 (8.09)	42 (12.4)	340 (7.76)	493 (7.29)
Between-meals snacking, n (%)		7723 (34.75)	135 (39.8)	1499 (34.23)	2331 (34.5)
Dietary supplement use, n (%)		4306 (19.37)	77 (22.7)	779 (17.79)	1152 (17.0)

**Table 2:** Baseline food consumption, energy and nutrient intakes, and dietary attitudes of the participants, of the Seguimiento Universidad de Navarra cohort, who filled out the paper-based and Web-based questionnaire at baseline and at 10-year follow-up.

Variable	Baseline questionnaires (Q_0)		10-year of follow-up questionnaires (Q_10)	
	Paper-based (n=22,225)	Web-based (n=339)	Paper-based (n=4379)	Web-based (n=6765)
Energy intake (kcal/d), mean (SD)	2532 (957)	2342 (882)	2566 (985)	2537 (904)
Protein intake (% total energy), mean (SD)	18.1 (3.5)	18.1 (4.7)	18.0 (3.7)	17.7 (3.3)
Carbohydrate intake (% total energy), mean (SD)	43.3 (7.7)	41.9 (10.6)	43.4 (8.0)	43.6 (7.3)
Fat intake (% total energy), mean (SD)	36.6 (6.8)	35.8 (8.9)	36.6 (7.1)	36.7 (6.5)
Polyunsaturated fatty acid intake (% total energy), mean (SD)	5.2 (1.6)	4.9 (1.7)	5.3 (1.7)	5.3 (1.6)
Monounsaturated fatty acid intake (% total energy), mean (SD)	15.7 (3.8)	15.3 (4.6)	15.8 (4.0)	15.7 (3.7)
Saturated fatty acid intake (% total energy), mean (SD)	12.5 (3.4)	11.6 (3.9)	12.5 (3.6)	12.6 (3.1)
Fiber intake (g/d), mean (SD)	30.0 (16.5)	30.6 (17.7)	30.7 (17.5)	28.8 (14.7)
Cholesterol intake (mg/d), mean (SD)	440.5 (208.7)	401.2 (183.2)	447.8 (218.5)	440.0 (185.1)
Alcohol intake (g/d), mean (SD)	6.8 (10.8)	5.1 (7.6)	6.9 (11.4)	7.0 (10.7)
Fruits (g/d), mean (SD)	370.9 (354.4)	353.9 (341.3)	380.6 (360.3)	349.1 (345.6)
Vegetables (g/d), mean (SD)	555.8 (409.8)	611.7 (533.0)	554.4 (446.0)	519.6 (336.1)
Nuts (g/d), mean (SD)	8.4 (16.6)	13.6 (27.3)	8.5 (16.5)	7.8 (16.1)
Legumes (g/d), mean (SD)	24.2 (25.2)	23.3 (17.9)	24.6 (25.7)	23.3 (20.8)
Dairy products (g/d), mean (SD)	209.4 (226.0)	136.1 (150.9)	222.3 (237.0)	231.1 (227.6)
Meats (g/d), mean (SD)	185.9 (105.4)	175.4 (94.2)	185.7 (110.0)	181.7 (92.9)
Fish (g/d), mean (SD)	103.0 (81.9)	100.3 (75.1)	104.8 (87.6)	98.2 (69.8)
Olive oil (g/d), mean (SD)	16.0 (14.5)	16.7 (16.1)	16.1 (15.1)	15.6 (14.3)
Fast food (g/d), mean (SD)	23.4 (27.5)	27.8 (23.6)	19.7 (25.9)	22.7 (29.0)
Do you try to eat more fiber? (% yes), n (%)	13153 (59.18)	223 (65.8)	2644 (60.38)	3820 (56.47)
Do you try to eat more fruit? (% yes), n (%)	15209 (68.43)	246 (72.6)	3059 (69.86)	4410 (65.19)
Do you try to eat more vegetables? (% yes), n (%)	17768 (79.95)	271 (80.0)	3551 (81.09)	5303 (78.39)
Do you try to eat more fish? (% yes), n (%)	13170 (59.26)	201 (59.3)	2644 (60.38)	3820 (56.47)
Do you avoid the consumption of butter? (% yes), n (%)	15649 (70.41)	247 (72.9)	3148 (71.89)	4574 (67.61)
Do you try to eat less fat? (% yes), n (%)	17312 (77.89)	270 (79.7)	3473 (79.31)	5079 (75.08)
Do you try to eat less meat? (% yes), n (%)	7747 (34.86)	145 (42.8)	1589 (36.29)	2205 (32.59)
Do you try to remove fat from meat? (% yes), n (%)	16549 (74.46)	187 (55.2)	3321 (75.84)	5064 (74.86)
Do you add sugar to some beverages? (% yes), n (%)	6712 (30.20)	99 (29.2)	1308 (29.87)	2125 (31.41)
Do you try to eat less sweets and pastries? (% yes), n (%)	14098 (63.43)	244 (72.0)	2720 (62.11)	3989 (58.87)

### Principal Results

We have found similar results to those previously published [32,33]. In Table 3, we provide the adjusted differences in the mean number of missing values in several items of the general questionnaire at baseline.

Generally, the adjusted differences in the 6 sections evaluated at Q_0 were low. In all cases, they were lower than 3.5%, even for questions on healthy eating attitudes, alcohol consumption, and personality traits; they were very low, lower than 0.5%. However, when we compared participants who completed Q_0 at baseline using the Web-based versus paper-based version, we found some contradictory data. Thus, the mean amount of missing data in the FFQ_0 and physical activity during leisure time questions was significantly higher among subjects who had completed the Web-based version (ß=3.3, 95% CI 2.03-4.64 and ß=2.01, 95% CI 1.45-2.57, respectively) but significantly lower in the other activity questions (ß=–2.01, 95% CI –3.04 to –1.35). Overall, the percentage of participants with implausible data on food consumption in the FFQ was always lower than 3% in participants who used the paper-based version at baseline or at 10-year follow-up (Table 4).

However, we found the following exception: the implausible reporting of nuts consumption, accounting for more than 16% of participants. On the other hand, when we considered all participants, generally, no significant differences among food group consumption were found, except for the implausible reporting of nuts consumption, more probable in these same subjects—odds ratio (OR) 1.54 (95% CI 1.20-1.99). When we classified participants according to the type of questionnaire chosen at 10-year follow-up, the implausible reporting of legume and meat consumption was less frequent in those who collected the Web-based version. The number of participants with implausible olive oil consumption was 1 among those who used the Web-based version and 0 among those who used the paper-based version. For this reason, we could not present the OR for Web-based versus paper-based version. Finally, overall, in both the paper-based and Web-based FFQ_0, the items with higher frequency of mismatches and inconsistencies were total energy intake (near 10%) and self-reported alcohol consumption (approximately 2%; Table 5).

On the contrary, mismatches and inconsistencies were lower in reported consumption of fruit dessert versus total fruit consumption and fried food versus oil consumption. In addition, subjects who filled the Q_0 via internet exhibited a significantly higher risk of presenting inconsistencies in reporting alcohol consumption and wine consumption versus total alcohol intake, compared with those who chose the paper-based version: adjusted OR 3.58 (95% CI 2.01-6.22) and OR 3.01 (95% CI 1.21-7.48), respectively, although the mismatches in relation to total energy intake outside of the reference subset had a lower risk: OR 0.31 (95% CI 0.17-0.56).

**Table 3:** Adjusted differences for the mean number of missing values in paper-based or Web-based questionnaire at baseline (Beta regression coefficients and 95% CIs). The Q_0 paper-based version was always considered as the reference category.

Missing values	All participants		Participants with successful 10-year follow-up	
	Missing values in baseline questionnaire^a^, mean (n=22,225)	Beta (95% CI)^b,c^ (n=339)	Missing values in 10-year of follow-up questionnaire^d^, mean (n=4379)	Beta (95% CI)^b,e^ (n=6765)
Missing values in the food-frequency questionnaire (136 items)	12.93	3.33 (2.03 to 4.64)^f^	14.54	–1.16 (–1.63 to –0.69)^f^
Missing values in the healthy eating attitudes (10 items)	0.29	0.13 (0.001 to 0.25)^g^	0.28	0.002 (–0.04 to 0.04)
Missing values in the alcohol consumption questions (5 items)	0.05	0.09 (0.05 to 0.12)^f^	0.07	–0.01 (–0.03 to 0.0002)
Missing values in the physical activity during leisure time questions (17 items)	2.99	2.01 (1.45 to 2.57)^f^	4.20	–0.21 (–0.43 to 0.01)
Missing values in the other activities questions (24 items)	4.60	–2.20 (–3.05 to –1.35)^f^	4.86	–0.45 (–0.74 to –0.16)^h^
Missing values in the personality traits questions (3 items)	0.04	0.07 (0.04 to 0.10)^f^	0.04	0.007 (–0.004 to 0.02)

^a^Q_0, paper-based.

^b^Adjusted for sex, age, level of education (bachelor, graduate, postgraduate, and doctorate) and year entering the cohort (1999-2000, 2001, 2002-2003, 2004, 2005-2007, and 2008-2017).

^c^In the mean number of missing values in baseline questionnaire (Q_0, Web-based -paper-based).

^d^Q_10, paper-based.

^e^In the mean number of missing values in 10-year of follow-up questionnaire (Q_10, Web-based -paper-based).

^f^*P*<.001.

^g^*P*<.05.

^h^*P*<.01.

**Table 4:** Percentage of participants with implausible report of food items in the paper-based or Web-based questionnaire at baseline. Odds ratios and 95% CIs to have implausible data in these dietary variables. The Q_0 paper-based version was always considered as the reference category.

Implausible report^a^	All participants		Participants with successful 10-year follow-up	
	Q_0^b^ Paper-based (n=22,225)	ORs^c^ (95% CI)^d^ for Web-based versus paper-based in Q_0 (n=339)	Q_10^e^ Paper-based (n=4379)	ORs (95% CI)^d^ for Web-based versus paper-based in Q_10 (n=6765)
Fruit consumption	1.77	0.49 (0.15-1.59)	1.99	0.77 (0.57-1.04)
Vegetable consumption	1.25	1.03 (0.44-2.43)	1.21	0.71 (0.48-1.06)
Legume consumption	2.84	1.07 (0.60-1.92)	2.88	*0.78 (0.61-0.99)* ^f^
Fish consumption	0.75	0.76 (0.18-3.22)	0.73	0.81 (0.49-1.32)
Meat consumption	0.62	0.33 (0.00-1.79)^g^	0.8	*0.32 (0.18-0.57)* ^h^
Dairy products consumption	1.09	0.19 (0.00-1.002)^g^	1.42	0.77 (0.54-1.09)
Cereal consumption	0.85	0.79 (0.19-3.37)	1.14	0.85 (0.57-1.27)
Nuts consumption	16.67	1.54 (1.20-1.99)^i^	16.4	0.94 (0.85-1.05)

^a^For each food group, the consumption was considered implausible if it fell outside the 25th percentile minus 3 times this interquartile range or 75th percentile plus 3 times this interquartile range.

^b^Q_0: Baseline questionnaire.

^c^OR: odds ratio.

^d^Adjusted for sex, age, level of education (bachelor, graduate, postgraduate, and doctorate), and year entering the cohort (1999-2000, 2001, 2002-2003, 2004, 2005-2007, and 2008-2017).

^e^Q_10: 10-year of follow-up questionnaire.

^f^*P*<.05.

^g^Exact logistic regression, as there was 0% of missing values in the Web-based questionnaire (unadjusted).

^h^*P*<.001.

^i^*P*<.01.

**Table 5:** Percentage of participants with mismatches and inconsistencies in dietary variables the paper-based or the Web-based questionnaire at baseline. Odds ratios and 95% CIs for presenting mismatches and inconsistencies in dietary variables.

Mismatches and inconsistencies in dietary variables	All participants		Participants with successful 10-year follow-up	
	Q_0^a^ (paper- based; n=22,225)	ORs^b^ (95% CI)^c^for Web-based versus paper-based in Q_0 (n=339)	Q_10^d^ (paper-based; n=4379)	ORs (95% CI)^c^ for Web-based versus paper-based in Q_10 (n=6765)
Total energy intake outside the predefined limits (Willett; <800 or >4000 kcal/d for men, <500 or >3500 kcal/d for women)	9.49	0.77 (0.54-1.13)	10.16	1.14 (0.99-1.30)
Total energy intake outside of the reference subset (<P1 or >P99)	1.96	*0.31 (0.17-0.56* **)^e^**	2.19	1.23 (0.93-1.63)
Total energy intake outside of the reference subset (<P5 or >P95)	9.96	0.73 (0.52-1.02)	10.46	1.11 (0.98-1.27)
Inconsistencies in reporting fruit dessert versus total fruit consumption	0.14	3.06 (0.36-26.0)	0.21	0.41 (0.14-1.18)
Inconsistencies in reporting fried food consumption versus oil consumption	0.81	1.63 (0.37-7.07)	1.14	0.70 (0.45-1.07)
Inconsistencies in reporting alcohol consumption	2.25	*3.58 (2.06-6.22)* ^e^	2.72	1.02 (0.79-1.30)
Inconsistencies in reporting wine consumption versus total alcohol intake	1.45	*3.01 (1.21-7.48)* ^f^	2.01	1.20 (0.89-1.62)

^a^Q_0: Baseline questionnaire.

^b^OR: odds ratio.

^c^Adjusted for sex, age, level of education (bachelor, graduate, postgraduate and doctorate), and year entering the cohort (1999-2000, 2001, 2002-2003, 2004, 2005-2007, and 2008-2017).

^d^Q_10: 10-year of follow-up questionnaire.

^e^*P*<.001.

^f^*P*<.05.

## Discussion

### Principal Findings

To the best of our knowledge, our research is the first to compare the quality of the answers, at baseline and after a 10-year follow-up period, in different subjects in a large prospective cohort, where some of them used the traditional version (paper-based) and others used a new method (Web-based) for data collection in a large prospective cohort. Our most important objective was to compare the respective ability of each method (Web-based or paper-based) to gather the relevant information in a reliable and comprehensive manner. The overall response rates in Q_0 were higher for the paper-based version than the Web-based version, 98.5% and 1.5%, respectively, as the electronic version of the questionnaire has only been available since 2004. In addition, the paper-based version was always offered as the first choice. However, in Q_10, the proportion of participants choosing the Web-based version was 61%, because of the fact that if participants are given a choice, they prefer the electronic version. A previous publication suggested that it could be useful to offer all subjects both a paper-based and a Web-based version of a long-term instrument, such as the FFQ, to avoid selection bias [34]. We found that in both methods, baseline characteristics, including food consumption and energy and nutrient intake, were comparable across a range of parameters, with a few exceptions. Although our present assessment does not represent a proper validation study, these findings are in agreement with previous studies that have shown few differences in health questionnaire scores or measures among different methods of administration [13,14]. For example, the NutriNet-Santé study published that the Web-based sociodemographic and economic questionnaire provided information of similar-to-superior quality compared with the traditional paper-based version [17]. Overall, the percentage of missing values in FFQ_0, with 136 items, was higher than 9%, whereas the missing information on physical activity during leisure time and activity questions, with 17 items, was higher than 17%, despite these questionnaires being shorter and simpler than the FFQ. On the other hand, although several studies in different areas have suggested that the validity and reliability of data obtained on the Web are comparable with those obtained by classical methods [35], our findings reveal that the quality of the data has not worsened with the incorporation of the Web-based version in the SUN cohort of highly educated adults. In fact, for some measures of data quality, the results have been more favorable among subjects who filled in questionnaires using the Web-based version, but for other measures, the data quality was worse. An exception is in relation to the implausible values of nut consumption, greater than 24% in FFQ_10. This is probably because of the fact that the majority of these participants reported their FFQ_10 after 2013, when the evidence on the health effects of this food group in the cardiovascular prevention of Primary Prevention of Cardiovascular Disease with a Mediterranean Diet trial had been published [36]. Moreover, the prominence of nuts in the latest published dietary guidelines has also contributed to the remarkable increase in their consumption. In addition, it is possible that SUN participants with longer follow-up were more aware of their diet because of general study participation and the administration of dietary recall methods, which resulted in improvements in their dietary habits (Hawthorne effect).

### Strengths and Limitations

The strengths of the SUN study are its high retention rate (91%) [25], a relatively long follow-up, its prospective design, the large sample, and its coordination in a single center, as well as the fact that many of the measurements have been validated. In addition, this study is a comparison and not a validation. Thus, we compared within the same study the self-reported Q_0 and Q_10 and the same FFQ_0 and the FFQ_10. Both FFQs had an identical format and did not incorporate photographs of serving sizes. For this reason, all answers to Q_O and Q_10 are most definitely comparable among themselves. We acknowledge that this study has several limitations. First, this study is not a validation of the same method of dietary assessment; rather, it has two different versions of assessment: paper-based or Web-based. In addition, the aim of this publication was not to compare repeated measurements of the same variables within the same subjects using 2 different methods. Consequently, our findings should be interpreted with caution, and they should never be analyzed as results of a validation study. Second, our sample of Web-based responders of FFQ_10 was small, with respect to the responders of the general Q_0. Thus, among participants with Q_10 (n=11,144), 6765 filled it out using internet, and among them, only 5882 completed the full-length FFQ_10, approximately 53% of participants with 10-year assessment. In addition, only 1.5% of Q_0 was filled out using the internet, and the results of the comparisons of this questionnaire, according to the form of administration, should be interpreted with caution. Third, Web-based cohorts were used to include motivated internet-skilled volunteers; this selection bias is not likely in our cohort, as there is wide access to the internet in all subgroups of the population [7]. On the other hand, despite a selection bias that could be the major factor limiting the generalizability of results, because of the nonrepresentative nature of the internet population, this bias is not very likely, as in Spain, 84.6% of the population between 16 and 74 years had easy access to the internet in the last 3 months [37]. However, it is possible that some older study participants who have greater difficulty using the computer did not fill out the Web-based questionnaires, as they needed a completion guide. Although in the SUN project all participants are university graduates with total access to internet and computers, without apparent difficulties of use, we could have hoped for higher response rates for the Web-based questionnaires [38]. Fourth, there was a difference in the way the Q_0 (paper) and the Q_10 (by internet) were filled out, which could have introduced a systematic error in our results. Although they contained exactly the same items and portions and only varied in format of presentation, it is possible that the previous knowledge of participants when they completed the follow-up questionnaire may have also interfered in their answers of follow-up questionnaires, but this bias affects both groups. Fifth, in the SUN cohort, if participants forget to click on an answer, an error message does not appear before they can go to the next page. For this reason, in some questions of Q_0 or Q_10 by internet, the average number of data missing is high. Sixth, this cohort was formed by graduates from University of Navarra, as well as from other different Spanish universities and professional associations, limiting the external validity of our results, which is required to extrapolate the present findings to the general population. However, epidemiology cohorts are usually nonrepresentative, and generalization should be based on biological plausibility. Seventh, participants could complete the Web-based questionnaires only since 2004, and this fact might result in underestimation of the effects of administration mode on the survey metrics. Finally, the SUN study is based on self-reported information; however, because of the high motivation of the participants (only 10% of the participants accepted to enter to the cohort) [39] and their high education level, we can assume high quality data. This paper provides an overview of the implementation of novel technologies in a large-sample cohort. The main lesson learned from methodological research in the context of the SUN cohort is the following: there is a need for the electronic version to be validated against the original paper-based format. On the other hand, the Web-based form should be designed to be completed in parts, should automatically reject incomplete questionnaires, and should also point out missing or contradictory items [35]. However, these messages may increase respondent frustration and thus decrease completion rates [40]. Minimal technical problems still need improvement before these new methods become common practice [41]. In conclusion, in the digital era, technological progress is having a significant impact on all aspects of our lives, and it is also accelerating scientific discoveries and changing research methods [2]. Several methods for the assessment of dietary intake are currently available in nutritional epidemiology, all have their own limitations and advantages. Thus, the dietary assessment methods should always be selected with caution, considering the research, objective, hypothesis, design, and available resources [10]. Finally, although the performance of innovative dietary assessment technologies has been investigated, more research is needed in regard to their validity and the most effective future strategies that could be incorporated into nutritional epidemiology [20,40,41].

## Abbreviations

FFQ: food-frequency questionnaire
FFQ_0: baseline semiquantitative food-frequency questionnaire
FFQ_10: 10 years of follow-up food-frequency questionnaire
MET: metabolic equivalent
OR: odds ratio
Q_0: baseline questionnaire
Q_10: 10-year of follow-up questionnaire
SUN: Seguimiento Universidad de Navarra

## References

[1] Boeing H (2013). Nutritional epidemiology: new perspectives for understanding the diet-disease relationship?. Eur J Clin Nutr.

[2] Brennan L, McNulty B (2017). New technology in nutrition research and practice. Proc Nutr Soc.

[3] van Gelder MM, Bretveld RW, Roeleveld N (2010). Web-based questionnaires: the future in epidemiology?. Am J Epidemiol.

[4] Illner A, Freisling H, Boeing H, Huybrechts I, Crispim SP, Slimani N (2012). Review and evaluation of innovative technologies for measuring diet in nutritional epidemiology. Int J Epidemiol.

[5] Hercberg S (2012). Web-based studies: the future in nutritional epidemiology (and overarching epidemiology) for the benefit of public health?. Prev Med.

[6] Amoutzopoulos B, Steer T, Roberts C, Cade JE, Boushey CJ, Collins CE, Trolle E, de Boer EJ, Ziauddeen N, van Rossum C, Buurma E, Coyle D, Page P (2018). Traditional methods new technologies-dilemmas for dietary assessment in large-scale nutrition surveys and studies: a report following an international panel discussion at the 9th International Conference on Diet and Activity Methods (ICDAM9), Brisbane, 3 September 2015. J Nutr Sci.

[7] Kesse-Guyot E, Assmann K, Andreeva V, Castetbon K, Méjean C, Touvier M, Salanave B, Deschamps V, Péneau S, Fezeu L, Julia C, Allès B, Galan P, Hercberg S (2016). Lessons learned from methodological validation research in E-epidemiology. JMIR Public Health Surveill.

[8] Touvier M, Kesse-Guyot E, Méjean C, Pollet C, Malon A, Castetbon K, Hercberg S (2011). Comparison between an interactive web-based self-administered 24 h dietary record and an interview by a dietitian for large-scale epidemiological studies. Br J Nutr.

[9] Benedik E, Koroušić Seljak B, Simčič M, Rogelj I, Bratanič B, Ding EL, Orel R, Fidler Mis N (2014). Comparison of paper- and web-based dietary records: a pilot study. Ann Nutr Metab.

[10] Shim JS, Oh K, Kim HC (2014). Dietary assessment methods in epidemiologic studies. Epidemiol Health.

[11] González Carrascosa R, García Segovia P, Martínez Monzó J (2011). Paper and pencil vs online self-administered food frequency questionnaire (FFQ) applied to university population: a pilot study. Nutr Hosp.

[12] Ngo J, Engelen A, Molag M, Roesle J, García-Segovia P, Serra-Majem L (2009). A review of the use of information and communication technologies for dietary assessment. Br J Nutr.

[13] Gwaltney CJ, Shields AL, Shiffman S (2008). Equivalence of electronic and paper-and-pencil administration of patient-reported outcome measures: a meta-analytic review. Value Health.

[14] Muehlhausen W, Doll H, Quadri N, Fordham B, O'Donohoe P, Dogar N, Wild DJ (2015). Equivalence of electronic and paper administration of patient-reported outcome measures: a systematic review and meta-analysis of studies conducted between 2007 and 2013. Health Qual Life Outcomes.

[15] Campbell N, Ali F, Finlay AY, Salek SS (2015). Equivalence of electronic and paper-based patient-reported outcome measures. Qual Life Res.

[16] Touvier M, Méjean C, Kesse-Guyot E, Pollet C, Malon A, Castetbon K, Hercberg S (2010). Comparison between web-based and paper versions of a self-administered anthropometric questionnaire. Eur J Epidemiol.

[17] Vergnaud AC, Touvier M, Méjean C, Kesse-Guyot E, Pollet C, Malon A, Castetbon K, Hercberg S (2011). Agreement between web-based and paper versions of a socio-demographic questionnaire in the NutriNet-Santé study. Int J Public Health.

[18] Ebert JF, Huibers L, Christensen B, Christensen MB (2018). Paper- or web-based questionnaire invitations as a method for data collection: cross-sectional comparative study of differences in response rate, completeness of data, and financial cost. J Med Internet Res.

[19] Smith B, Smith T, Gray G, Ryan MA, Millennium Cohort Study Team (2007). When epidemiology meets the internet: web-based surveys in the millennium cohort study. Am J Epidemiol.

[20] Mikkelsen EM, Hatch E, Wise L, Rothman K, Riis A, Sørensen HT (2009). Cohort profile: the Danish web-based pregnancy planning study--'Snart-Gravid'. Int J Epidemiol.

[21] Knudsen VK, Hatch EE, Cueto H, Tucker KL, Wise L, Christensen T, Mikkelsen EM (2016). Relative validity of a semi-quantitative, web-based FFQ used in the 'Snart Forældre' cohort - a Danish study of diet and fertility. Public Health Nutr.

[22] Wise LA, Rothman KJ, Mikkelsen EM, Stanford JB, Wesselink AK, McKinnon C, Gruschow SM, Horgan CE, Wiley AS, Hahn KA, Sørensen HT, Hatch EE (2015). Design and conduct of an internet-based preconception cohort study in North America: pregnancy study online. Paediatr Perinat Epidemiol.

[23] Lind L, Elmståhl S, Bergman E, Englund M, Lindberg E, Michaelsson K, Nilsson PM, Sundström J (2013). EpiHealth: a large population-based cohort study for investigation of gene-lifestyle interactions in the pathogenesis of common diseases. Eur J Epidemiol.

[24] Conrad J, Nöthlings U (2017). Innovative approaches to estimate individual usual dietary intake in large-scale epidemiological studies. Proc Nutr Soc.

[25] Carlos S, De La Fuente-Arrillaga C, Bes-Rastrollo M, Razquin C, Rico-Campà A, Martínez-González MA, Ruiz-Canela M (2018). Mediterranean diet and health outcomes in the SUN cohort. Nutrients.

[26] Martin-Moreno J, Boyle P, Gorgojo L, Maisonneuve P, Fernandez-Rodriguez JC, Salvini S, Willett WC (1993). Development and validation of a food frequency questionnaire in Spain. Int J Epidemiol.

[27] Fernández-Ballart JD, Piñol JL, Zazpe I, Corella D, Carrasco P, Toledo E, Perez-Bauer M, Martínez-González MA, Salas-Salvadó J, Martín-Moreno JM (2010). Relative validity of a semi-quantitative food-frequency questionnaire in an elderly Mediterranean population of Spain. Br J Nutr.

[28] Mataix VJ (2003). [Spanish Food Composition Table].

[29] Moreiras O, Carbajal A, Cabrera L (2005). Tablas de composición de alimentos (Food Composition Tables), 9th ed.

[30] Willett W (1998). Issues in analysis and presentation of dietary data. Nutritional Epidemiology, 2nd edition.

[31] Martínez-González MA, López-Fontana C, Varo JJ, Sánchez-Villegas A, Martinez JA (2005). Validation of the Spanish version of the physical activity questionnaire used in the Nurses' Health Study and the Health Professionals' Follow-up Study. Public Health Nutr.

[32] Andrade L, Zazpe I, Santiago S, Carlos S, Bes-Rastrollo M, Martínez-González MA (2017). Ten-year changes in healthy eating attitudes in the SUN cohort. J Am Coll Nutr.

[33] Santiago S, Zazpe I, Gea A, de la Rosa PA, Ruiz-Canela M, Martínez-González MA (2017). Healthy-eating attitudes and the incidence of cardiovascular disease: the SUN cohort. Int J Food Sci Nutr.

[34] Illner AK, Harttig U, Tognon G, Palli D, Salvini S, Bower E, Amiano P, Kassik T, Metspalu A, Engeset D, Lund E, Ward H, Slimani N, Bergmann M, Wagner K, Boeing H (2011). Feasibility of innovative dietary assessment in epidemiological studies using the approach of combining different assessment instruments. Public Health Nutr.

[35] Eysenbach G, Wyatt J (2002). Using the internet for surveys and health research. J Med Internet Res.

[36] Estruch R, Ros E, Salas-Salvadó J, Covas M, Corella D, Arós F, Gómez-Gracia E, Ruiz-Gutiérrez V, Fiol M, Lapetra J, Lamuela-Raventos RM, Serra-Majem L, Pintó X, Basora J, Muñoz MA, Sorlí JV, Martínez JA, Fitó M, Gea A, Hernán MA, Martínez-González MA, PREDIMED Study Investigators (2018). Primary prevention of cardiovascular disease with a Mediterranean diet supplemented with extra-virgin olive oil or nuts. N Engl J Med.

[37] The National Statistics Institute.

[38] Greenlaw C, Brown-Welty S (2009). A comparison of web-based and paper-based survey methods: testing assumptions of survey mode and response cost. Eval Rev.

[39] Naska A, Lagiou A, Lagiou P (2017). Dietary assessment methods in epidemiological research: current state of the art and future prospects. F1000Res.

[40] Lo Siou G, Csizmadi I, Boucher BA, Akawung AK, Whelan HK, Sharma M, Al Rajabi Ala, Vena JE, Kirkpatrick SI, Koushik A, Massarelli I, Rondeau I, Robson PJ (2017). The comparative reliability and feasibility of the past-year Canadian diet history questionnaire II: comparison of the paper and web versions. Nutrients.

[41] Christensen T, Riis AH, Hatch EE, Wise LA, Nielsen MG, Rothman KJ, Toft Sørensen H, Mikkelsen EM (2017). Costs and efficiency of online and offline recruitment methods: a web-based cohort study. J Med Internet Res.

